# HIF-1α Promotes Epithelial-Mesenchymal Transition and Metastasis through Direct Regulation of ZEB1 in Colorectal Cancer

**DOI:** 10.1371/journal.pone.0129603

**Published:** 2015-06-09

**Authors:** Wenjing Zhang, Xinpeng Shi, Ying Peng, Meiyan Wu, Pei Zhang, Ruyi Xie, Yao Wu, Qingqing Yan, Side Liu, Jide Wang

**Affiliations:** 1 Guangdong Provincial Key Laboratory of Gastroenterology, Department of Gastroenterology, Nanfang Hospital, Southern Medical University, Guangzhou, China; 2 Department of Medical Oncology, The First people’s Hospital of Yunnan Province, Kunming University of Science and Technology, Kunming, China; National Cancer Center, JAPAN

## Abstract

It is well recognized that hypoxia-inducible factor 1 alpha (HIF-1α) is involved in cancer metastasis, chemotherapy and poor prognosis. We previously found that deferoxamine, a hypoxia-mimetic agent, induces epithelial-mesenchymal transition (EMT) in colorectal cancer. Therefore, here we explored a new molecular mechanism for HIF-1α contributing to EMT and cancer metastasis through binding to ZEB1. In this study, we showed that overexpression of HIF-1α with adenovirus infection promoted EMT, cell invasion and migration *in vitro* and *in vivo*. On a molecular level, HIF-1α directly binding to the proximal promoter of ZEB1 via hypoxia response element (HRE) sites thus increasing the transactivity and expression of ZEB1. In addition, inhibition of ZEB1 was able to abrogate the HIF-1α-induced EMT and cell invasion. HIF-1α expression was highly correlated with the expression of ZEB1 in normal colorectal epithelium, primary and metastatic CRC tissues. Interestingly, both HIF-1α and ZEB1 were positively associated with Vimentin, an important mesenchymal marker of EMT, whereas negatively associated with E-cadherin expression. These findings suggest that HIF-1α enhances EMT and cancer metastasis by binding to ZEB1 promoter in CRC. HIF-1α and ZEB1 are both widely considered as tumor-initiating factors, but our results demonstrate that ZEB1 is a direct downstream of HIF-1α, suggesting a novel molecular mechanism for HIF-1α-inducing EMT and cancer metastasis.

## Introduction

Epithelial-mesenchymal transition (EMT) is a crucial event in cancer metastasis, during which polarized epithelial cells are inclined to obtain certain characters of mesenchymal cells, as well as more migration and invasive properties [1]. The molecular hallmarkers during EMT include down-regulated epithelial markers (e.g., E-cadherin, plakoglobin and desmoplakin), up-regulated mesenchymal markers (e.g., Vimentin, N-cadherin and α-smooth muscle actin) and increased expression of transcription factors such as Snail, Slug, Twist, zinc finger E-box binding homeobox 1 (ZEB1), ZEB2, and/or E47, which can bind to E-cadherin promoter and inhibit its transcription activity and expression [2]. Notably, the loss or downregulation of E-cadherin is considered to be the primary and most important step of EMT. E-cadherin can be silenced by different mechanisms including aberrant methylation and transcriptional suppression. Among them, its transcription regulation is widely studied in various of malignant tumors [3].

Hypoxia-inducible factor 1 alpha (HIF-1α) has been reported to promote EMT in several types of tumors through modulating one or more EMT-associated genes [4]. As a transcription factor, HIF-1α regulates the activities of its downstream genes through binding the hypoxia response element (HRE) in their promoter regions. For example, HIF-1α is able to transactivate matrix metallopeptidase 9 (MMP9) in breast cancer [5]. In hepatocellular carcinoma, it activates Snail thus repressing E-cadherin expression [6]. Moreover, HIF-1α competes with transcription factor 4 (TCF4) for direct binding to β-catenin thereby enhancing EMT in colorectal cancer [7]. Hence, there exists a close link between EMT and HIF-1α expression in cancer with the mechanisms unknown.

ZEB1 is a crucial transcriptional factor of EMT [8–10]. However, the association between HIF-1α and ZEB1 is little known. In this study, we showed that HIF-1α overexpression induced EMT and metastatic phenotypes in CRC. HIF-1α directly regulated ZEB1 expression through the hypoxia response element 3 (HRE-3) which is located in the ZEB1 proximal promoter. Suppression of ZEB1 reversed these effects induced by HIF-1α overexpresssion. The expression profiles of HIF-1α, ZEB1 and Vimentin were much similar in CRC patients, which was opposite to E-cadherin expression. These results indicate that CRC progression and metastasis, induced by HIF-1α, is mediated by the direct regulation of ZEB1.

## Materials and Methods

### Cells and clinical specimens

CRC cell lines HT29 and HCT116 were maintained in our laboratory, under the condition with RPMI 1640 (GIBCO) supplemented with 10% fetal bovine serum (FBS) as described previously [26]. Human CRC and the according adjacent normal tissues were obtained from ten patients with CRC who underwent colonoscopy; human CRC and the matched metastatic lymph node tissues were obtained from thirty-two CRC patients who underwent hemicolectomy in Nanfang Hospital (Guangzhou, China). These individuals gave us their written informed consent (as outlined in the PLOS consent form) to participate in this study and publish these case details. The studies using human tissue were reviewed and approved by the Committees for Ethical Review of Research involving Human Subjects in Nanfang Hospital, Southern Medical University (Permit Number: NFYY-2012-75).

### Construction and production of recombinant adenovirus

HIF-1α-expressing plasmid, pDC316-HIF-1α-EGFP, was constructed by inserting the full-length HIF-1α cDNA into the restriction sites between NheI and NotI endonucleases of the pDC316 expression vector which contained EGFP (pDC316-EGFP). For stable knockdown of ZEB1, the following sequences were cloned into pDC316-HIF-1α-EGFP. shZEB1 forward: 5'-GATCCCCAGATGATGAATGCGAGTCGttcaagagaTGACTCGCATTCATCATCTTTTTTGGAAA-3' and shZEB1 reverse: 5'-AGCTTTTCCAAAAAAGATGATGAATGCGAGTCAtctcttgaaCGACTCGCATTCATCATCTGGG-3' as described previously [8]. Both plasmids were verified by DNA sequencing.

All adenoviruses, including adenovirus 5 expressing HIF-1α (Ad5-HIF-1α), HIF-1α overexpression and ZEB1 knockdown (Ad5-HIF-1α-shZEB1) and the control, Ad5-EGFP, were generated using AdMax adenovirus packaging system (Microbix Biosystems Inc., Ontario, Canada) according to the manufacturer’s instructions. Large-scale amplification of the above adenoviral vectors were conducted in HEK293T cells by homologous recombination between a shuttle plasmid (pDC316) and a backbone plasmid (pBHGlox_E1, 3Cre). Titres of the purified viruses were determined by standard plaque-forming assay according to the manufacturer’s instructions (Virapur, San Diego, CA).

### Adenovirus infections in vitro

HT29 and HCT116 cells were grown to 80% confluency. After washing with phosphate buffered saline (PBS), cells were incubated with Ad5-EGFP and Ad5-HIF-1α at the MOI of 0.1, 1, 10, 100 and 1000, respectively. Ninety minutes post-infection, viruses were replaced by regular growth medium. 24 or 48 hours post-infection, the efficiency of transduction of EGFP expressing constructs was observed under immunofluorescence microscopy and HIF-1α expression was analyzed by PCR or western blot.

### Reverse transcription polymerase chain reaction (RT-PCR), Quantative real-time PCR (qPCR) and Western Blotting Analysis

These assays were done essentially as described previously [27–29]. Primers used for RT-PCR were as follows: HIF-1α sense: 5’-TCCATGTGACCATGAGGAAA-3’ and antisense: 5’- CCAAGCAGGTCATAGGTGGT-3’; primers used for qPCR were as follows: HIF-1α: 5′- TTTTTCAAGCAGTAGGAATTGGA -3′ (sense) and 5′-GTGATGTAGTAGCTGCATGATCG -3′ (antisense); ZEB1: 5′-CCTGTCCATATTGTGATAGAGGC-3′ (sense) and 5′-ACCCAGACTGCGTCACATGT-3′ (antisense); glyceraldehyde-3-phosphate dehydrogenase (GAPDH): 5′-GTCAACGGATTTGGTCGTATTG-3′ (sense) and 5′- CTCCTGGAAGATGGTGATGGG-3′ (antisense). The primary antibodies, HIF-1α (Novus Biologicals, Littleton, CO), ZEB1 (Santa cruz), E-cadherin (Santa cruz), Vimentin (prediluted, abcam), Plakoglobin (abcam), N-cadherin (Santa cruz) and GAPDH (abcam) were all commercial products.

### Migration, invasion and wound healing assays

Uncoated Costar transwells (Corning Costar Co., Corning, NY) were used for migration assays and Matrigel-coated transwells (BD Biosciences, Franklin Lakes, NJ) used for invasion assays. Cells were serum starved overnight and then seeded into the upper chamber with serum-free RPMI 1640 medium. RPMI 1640 supplemented with 10% FBS was added into the lower chamber. Cells that had migrated across the transwell membrane were stained and quantified. For wound healing assay, the scratch was made across the cell monolayer using a sterile tip. The ability of cells to migrate was monitored at different time points using a light microscopy.

### Histological and immunohistochemical analysis

Hematoxylin and eosin (H&E) and immunohistochemistry (IHC) were performed as previously described [28]. In brief, specimens were fixed in 4% paraformaldehyde in PBS overnight and subsequently embedded in paraffin wax. Sections were cut a thickness of 5 μm and stained with H&E for histological analysis. IHC analysis was performed for the expression of HIF-1α, ZEB1, E-cadherin and Vimentin. The tissue in which >10% of cancer cells being positively stained was considered as positive. For quantitative analysis, the ratio of positively stained cells to all tumor cells in five random areas at 200-fold magnification was calculated. All histological evaluations including the percentage of positive cells were carried out in a double-blind manner by two pathologists to minimize observational bias.

### Electrophoretic Mobility Shift Assay (EMSA)

EMSA was performed using a double-stranded oligonucleotide containing a consensus binding sequence for HIF-1α as previously described [27, 28]. The following sequences of sense strands were used for the binding and competition assays: sequences containing wildtype HRE (P1: 5’- GAGGCGTGGGACTGATGGTAGCC -3’, -521 ~ -517 nt; P2: 5’- GGGGGCGGACACGCGAGG -3’ -529 ~ -525 nt; P3: 5’-CCGGTCGCCGCGTGTCCTCGCC -3’, -634 ~ -630 nt; P4: 5’-ATACTCCGGTCACGTTTCAGTTTTCTC -3’, -1347 ~ -1342 nt) and the according mutant HRE sequences (P1-mut: 5’- GAGGCACAGGACTGATGGTAGCC -3’; P2-mut: 5’-GGGGGCGGATGTGCGAGG -3’; P3-mut: 5’- CCGGTCGCCGCACATCCTCGCC -3’ and P4-mut: 5’- ATACTCCGGTTGTGTTTCAGTTTTCTC -3’). The nucleotides were end-labeled with [γ-32P] ATP (PerkinElmer Life and Analytical Sciences, Fremont, CA) and T4 polynucleotide kinase (Promega). Nuclear extracts from cells infected with Ad5-HIF-1α-EGFP and the control were prepared and used in EMTS as we described previously [28]. And were incubated in 1× binding buffer containing 2.5% glycerol, 50 ng/μl poly (dI-dC), 5 mm MgCl2, 0.05% Nonidet P-40, and 4 pmol of biotin-labeled oligonucleotide in a total volume of 20 μl at room temperature for 20 min. The bound mixtures were size-fractionated on a nondenaturing 6% polyacrylamide gel at 180 V in 0.5× TBE buffer. The gel was subsequently dried and autoradiographed.

### Generation of report plasmids, Transient transfection and Luciferase assay

Production of wildtype and mutant reporter constructs was performed as we previously described [29]. A 567 bp of ZEB1 promoter fragment containing the HRE-3 element was cloned into pGL3 vector to generate the wildtype reporter construct, named as pluc-567. Mutation at the HRE-3 motifs (pluc-567-mut) was introduced into pluc-567 luciferase construct with the QuikChange Site-Directed Mutagenesis Kit (Stratagene, La Jalla, CA, USA). Both of the constructs were verified by sequencing. Cells were infected with Ad5-EGFP or Ad5-HIF-1α for 48 h followed by transfection with pluc-567 or pluc-567-mut and pRL-cmv (*Renilla* luciferase). The transactivation potential was tested with the Dual-Glo Luciferase Assay System (Promega, Madison, WI, USA) by measuring luciferase activity after 48h.

### Nude mice and metastasis assay

This animal experiment was carried out in strict accordance with the recommendations in the Guide for the IACUC (Institutional Animal Care and Use Committee), and the protocol was approved by the Committee on the Ethics of Animal Experiments of Nanfang Hospital (Permit Number: NFYY-2013-56). All surgeries were performed under sodium pentobarbital anesthesia, and all efforts were made to minimize suffering. After the surgery, all nude mice were euthanized by sodium pentobarbital anesthesia. Six-week old female BALB/c nude mice were purchased from Guangdong Provincial Experimental Animal Center (Guangzhou, China) and randomly divided into four groups (Ad5-EGFP: N = 8; Ad5-HIF-1α: N = 12; Ad5-HIF-1α-shRNA: N = 5; Ad5-HIF-1α-shZEB1: N = 5) before injection. Animals were subjected into intraperitoneal anaesthesia with 40 mg/kg amobarbital sodium solution. After the mice were deeply anaesthetized, a small longitudinal left upper flank incision was made and spleen was gently exposed. Single suspended cells (1.5 × 106 cells/mouse) were injected in 70 μl of PBS under the spleen capsule. After removal of the needle, the injection site was pressed with an aseptic cotton sponge to prevent leakage. After that, the spleen was returned into abdominal cavity and the periotneium and abdominal wall were sutured with silk [30–32]. Mice were sacrificed after 5 weeks, the possibility of metastasis analyzed and the metastatic sites were collected for H&E staining and qPCR assay.

### Statistical analysis

Analysis was done using GraphPad Prism 6 software. All values were expressed as mean ± SD. The statistical significance of differences was determined by Student’s *t*-test. All analyses were two-sided, paired (for IHC results in clinical specimens) or unpaired and a *P* value of < 0.05 was considered statistically significant.

## Results

### HIF-1α overexpression induced EMT in vitro

**Fig 1:**
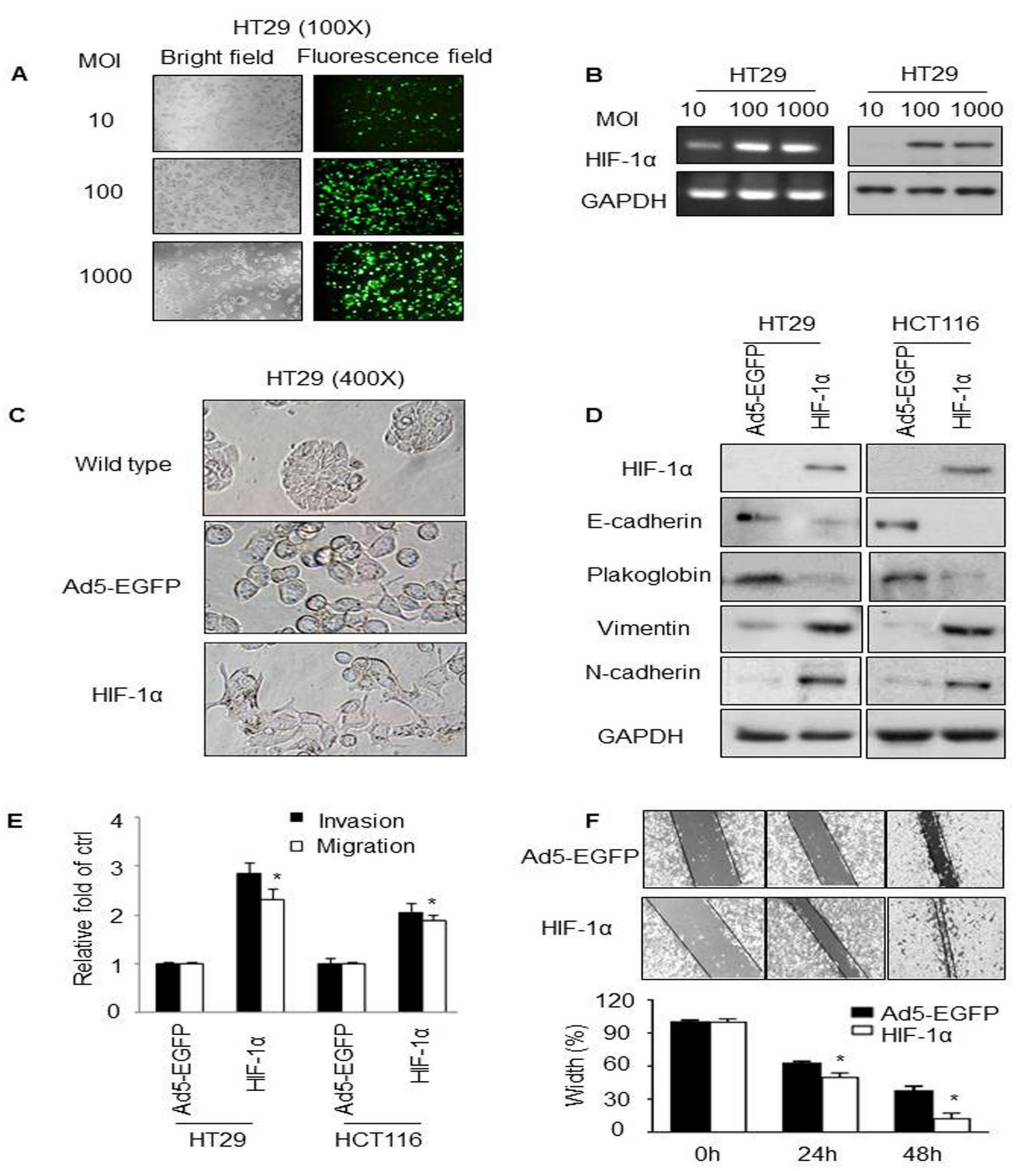
Overexpression of HIF-1α induced EMT and metastasis in CRC cell lines. (A) HT29 cells were transduced with Ad5-HIF-1α at the indicated MOI values for 48 hours. Fluorescence intensity and bright field at the same area were observed at the original magnification as 100X. (B) mRNA (left) and protein (right) expressions of HIF-1α were detected using RT-PCR and western blot, respectively. GAPDH was used as the loading control. (C) Cell morphology of wildtype HT29 or HT29 cells transduced with the indicated adenoviruses (Original magnification = 400X). (D) Western blot analysis of HIF-1α, E-cadherin, Plakoglobin, Vimentin and N-cadherin in adenovirus-infected HT29 and HCT116 cells. GAPDH was used as the internal control. (E) Fold change of invasion and migration in the indicated cells. Quantification of the results was shown in the bar graph with means ± SD. *P < 0.05. (F) Wound healing assay. Cell monolayers were scratched with a pipette tip and images were taken 0, 24 and 48 hours after wound formation. *P < 0.05.

To get a better infection efficacy of adenoviruses in CRC cancer cell lines, HT29 and HCT116 cells were first transduced with adenovirus 5 expressing enhanced green fluorescent protein (Ad5-EGFP) or Ad5-HIF-1α-EGFP at the multiplicity of infection (MOI) of 10, 100 and 1000, respectively, with the effects recorded by microscopy on 48 hours (Fig 1A). Nearly 100% of the cells expressed EGFP with a MOI of 100, which was much similar to a MOI of 1000. RT-PCR (Fig 1B left) and western blot (Fig 1B right) results confirmed the dramatic increase of HIF-1α expression. Same effect was found in HCT116 cells (data not shown). Therefore, the MOI of 100 was adopted in all of the following experiments.

Interestingly, when observing adenovirus-infected cells, we found HT29-Ad5-HIF-1α cells were much more spindle-shaped, like fibrobalsts, compared with wildtype HT29 cells which were round with well cell-cell adhesion (Fig 1C). The above transition of cell morphology actually was as the same as the change in EMT process. Hence, we next detected the expressions of EMT markers and found that E-cadherin and Plakoglobin, two important epithelial markers of EMT, were significantly downregulated in Ad5-HIF-1α-infected HT29 and HCT116 cells compared to their control cells. On the contrary, the mesenchymal molecules, Vimentin and N-cadherin were sharply increased after Ad5-HIF-1α-EGFP infection (Fig 1D). Furthermore, an increase in invasion and migration (crucial traits of EMT phenotype) of both cell lines with HIF-1α overexpression was also detected (Fig 1E). Consistently, the wound healing assay demonstrated that the width in HT29-Ad5-HIF-1α cells were much narrower than that in HT29-Ad5-EGFP cells at the indicated time plots, respectively (Fig 1F). The above findings suggest that HIF-1α overexpression induces EMT and promotes invasion and migration in CRC cell lines.

### HIF-1α overexpression promoted metastasis in vivo

**Fig 2:**
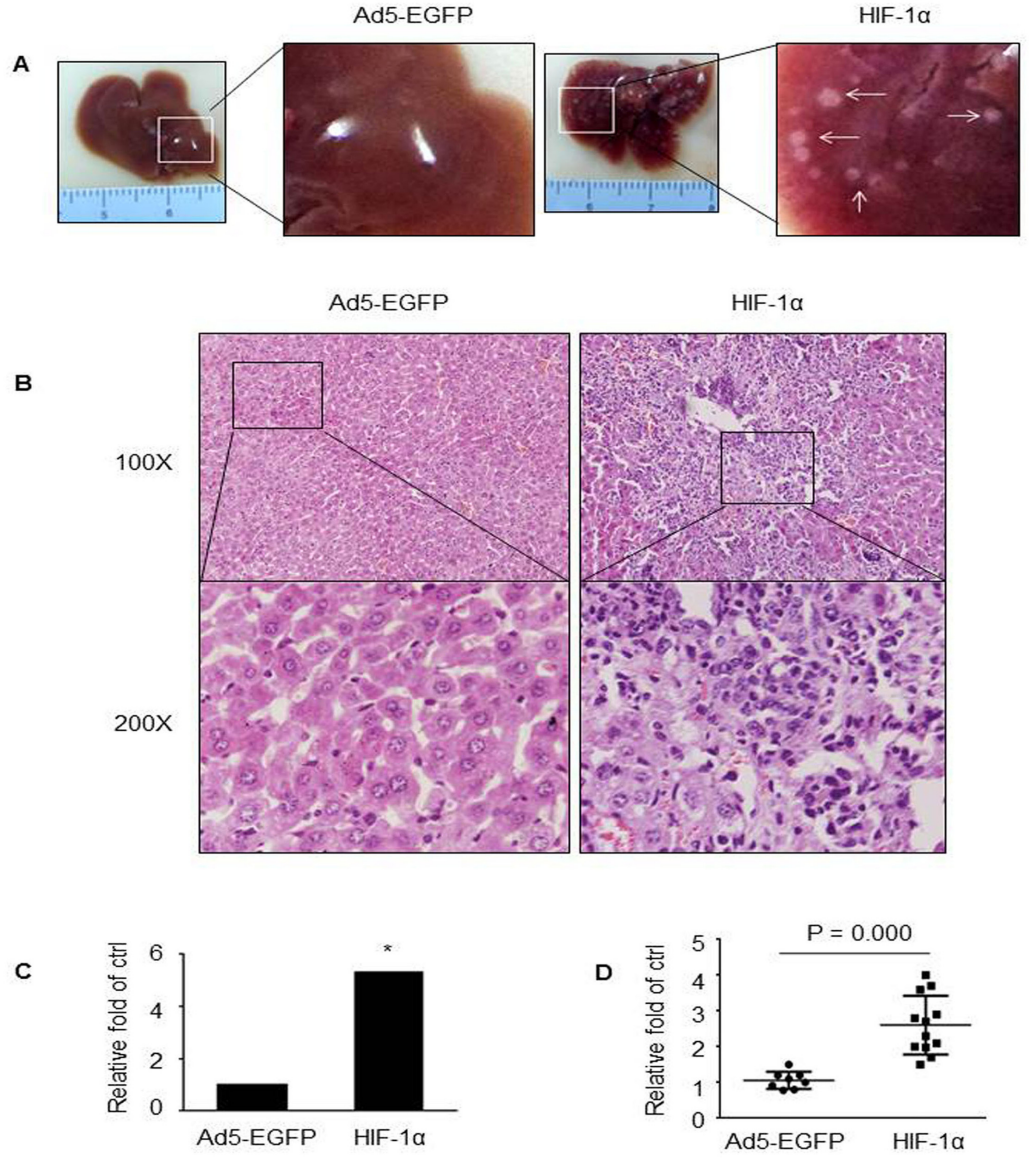
HIF-1α overexpression promoted liver metastasis *in vivo*. Representative photographic pictures (A) and H&E staining (B) of liver of BALB/c mice five weeks after subsplenic injection of HT29 cells transduced with Ad5-HIF-1α or control viruses. White arrows indicated metastatic nodules. (C) The comparison of liver metastases between HT29-Ad5-HIF-1α mice (N = 12) and control mice (N = 8). *P < 0.05. (D) Confirmation of the overexpression of HIF-1α from metastatic nodules in mice injected with Ad5-HIF-1α by qPCR, compared with those injected with control viruses.

To evaluate the effect of HIF-1α overexpression on cancer metastasis *in vivo*, HT29 cells were transduced with Ad5-HIF-1α-EGFP or Ad5-EGFP, respectively, at MOI of 100. Forty-eight hours later, single cell suspensions were harvested and injected into BALB/c nude mice via a subsplenic method. As shown in fig 2A, multiple intrahepatic tumor nodules were easily inspected grossly in the Ad5-HIF-1α-EGFP-injected group, whereas less or even no nodules were found in control mice. To confirm the above difference, we examined H&E staining and found much more developed basophilic tumor regions in livers from mice injected with HT29-Ad5-HIF-1α cells compared with control group, as shown in fig 2B. Quantity analysis showed that a significant increase (5.3-fold) of liver metastasis was noted in the Ad5-HIF-1α-injected group, compared with control group (Fig 2C). Real-time PCR results confirmed that the level of HIF-1α in liver lesions of mice injected with HT29-Ad5-HIF-1α was much higher than that injected with HT29-Ad5-EGFP (Fig 2D). These results suggest that overexpression of HIF-1α promotes liver metastasis of CRC in animal model.

### HIF-1α regulated ZEB1 expression

**Fig 3:**
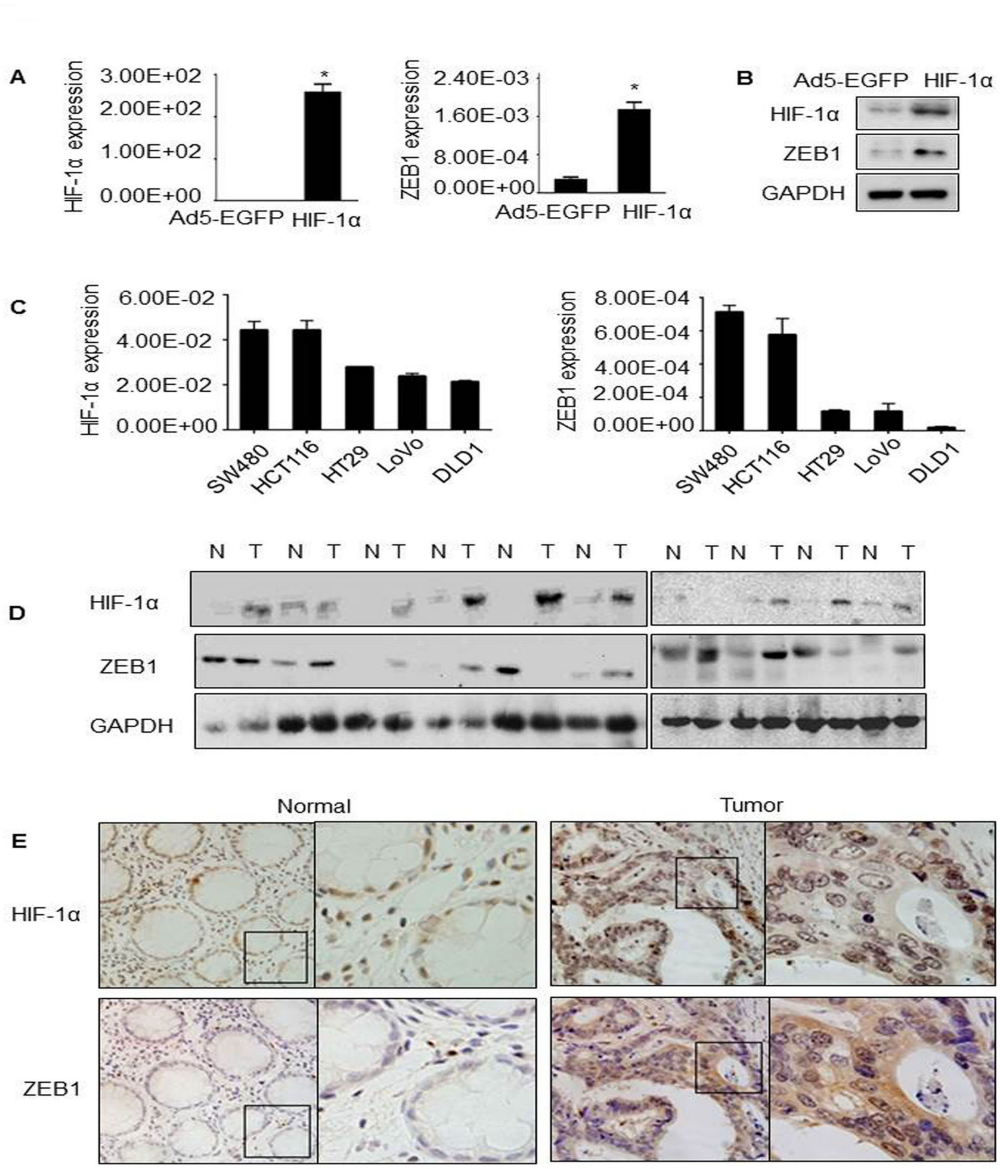
Regulation of HIF-1α on ZEB1 and their expressions in CRC. (A) Endogenous expression levels of HIF-1α and ZEB1 in the indicated five cell lines were detected by qPCR with GAPDH used as the internal control. (B&C) HT29 cells were transduced with Ad5-HIF-1α or control viruses for 48 hours, following by qPCR and western blot to detect the expression of HIF-1α and ZEB1. GAPDH was used as the internal control. (D) HIF-1α and ZEB1 protein levels in matched nonneoplastic/cancerous colorectal tissues. N: normal; T: tumor. The level of each protein was normalized against GAPDH. (E) Representative pictures of IHC staining on human CRC formalin-fixed paraffin-embedded samples for HIF-1α and ZEB1. Left panel showed the adjacent normal colorectal tissues. Right panel showed CRC specimens. Original magnification, 200X.

Both HIF-1α and ZEB1 have been implicated in cancer metastasis and EMT [4, 9, 11]. Next, we investigated whether HIF-1α overexpression had an effect on the expression of ZEB1. Indeed, upregulation of mRNA and protein levels of ZEB1 was found accompanied with the overexpression of HIF-1α in HT29 cells as shown in Fig 3A&3B. Moreover, the trends of HIF-1α and ZEB1 expression in CRC cell lines (SW480, HCT116, HT29, LoVo and DLD1) were pretty similar (Fig 3C). Consistently, this finding was also presented in paired CRC specimens and adjacent normal colon epithelium tissues which were detected by western blot and IHC staining (Fig 3D&3E). We also found that the levels of both proteins were much higher in tumor than that in adjacent normal tissues. In cellular level, both HIF-1α and ZEB1 were mainly expressed in nucleus and cytoplasm, especially in nucleus, which were theoretically consistent with the location site of transcription factor. And also, the observations of their co-localization reveal that HIF-1α might interact with ZEB1 physically.

### Regulation of ZEB1 by HIF-1α through HRE-3

**Fig 4:**
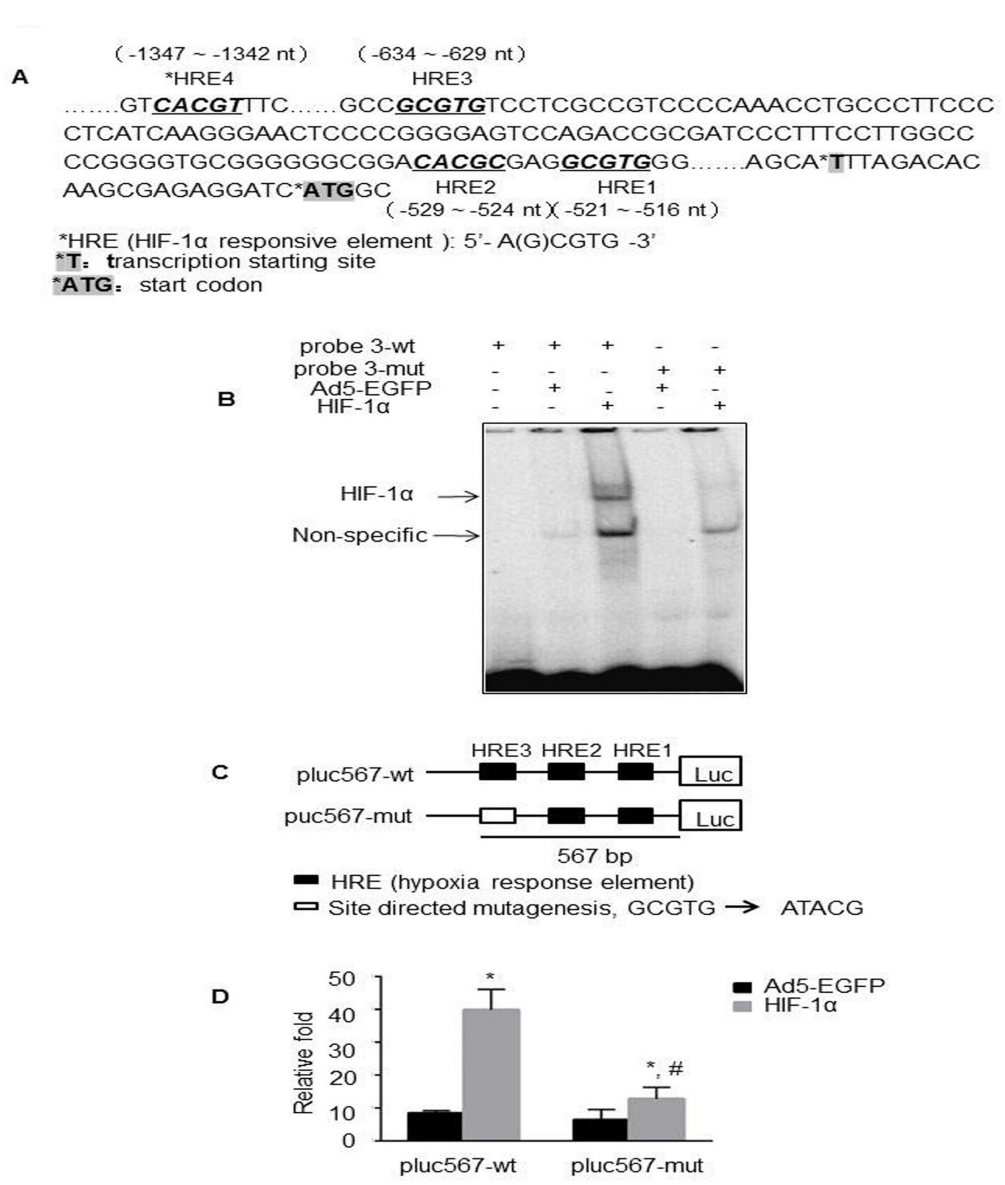
Regulation of ZEB1 by HIF-1α through HRE-3. (A) Schematic representation of the proximal promoter (~ 3500 nt upstream) of ZEB1 gene. HRE: hypoxia response element. (B) Oligonucleotides for EMSA were wildtype (lane 1–3) and mutant (lane 4–5) probe 3 from the ZEB1 promoter, which contained a consensus HRE-3. Nuclear extracts prepared from HT29-Ad5-HIF-1α (lane 3 and 5) or control cells (lane 2 and 4) were incubated with [γ-32P] ATP-labelled probe before electrophoresis. Negative control was performed with wildtype probe without nuclear extract (lane 1). (C) Schematic representation of the promoter region of ZEB1 and the report constructs used in adenovirus-infected experiments. The constructs contained wildtype (pluc567-wt) or mutant (pluc567-mut) HRE-3 located -634 ~ -630 nt upstream of the transcription start site of ZEB1. (D) Activation of plu567 or pluc567-mut in Ad5-HIF-1α- or Ad5-EGFP-infected HT29 cells (N = 3 replicate experiments). *, ^#^ *P* < 0.05.

To evaluate whether ZEB1 is regulated directly by HIF-1α, the promoter sequences of ZEB1 were analyzed with bioinformatics methods. We found that there were four potential HRE sites in the proximal promoter (~ 3500 nt upstream; the start codon ATG defined as 0) of ZEB1 gene (Fig 4A). There were HRE-1 at -517 ~ -521 nt; HRE-2 at -525 ~ -529 nt; HRE-3 at -630 ~ -634 nt and HRE-4 at -1342 ~ -1347 nt. Based on these, probes P1, P2, P3 and P4 with wildtype and mutant HRE sites were designed, respectively, as described in methods and materials. The results of EMSA assay revealed that HIF-1α-binding was significantly increased after incubation of nuclear extracts from HT29-Ad5-HIF-1α cells with the HRE-3-containing oligonucleotide from ZEB1 promoter (Fig 4B). However, there were only very week bands or even no band presented in HT29-Ad5-HIF-1α cells or other control cells with the HRE-1, 2 or 4-containing oligonucleotide (data not shown). Moreover, competition for HIF-1α-binding by unlabelled oligonucleotides containing HRE-3 and probes containing mutated HRE-3 did not show any HIF-1α-binding bands (Fig 4B). These results suggested that HIF-1α was able to bind ZEB1 promoter via HRE-3 site.

Next, we determined the effect of HIF-1α overexpression on the transcription activity of ZEB1. Based on the results of EMSA, a promoter plasmid containing HRE1-3 (pluc567) and the site-directed mutagenesis of HRE-3 plasmid (pluc567-mut) were generated and transiently transfected into HT29 cells which were pre-incubated with Ad5-HIF-1α or Ad5-EGFP for 24 h, Dual-luciferase assay showed that the activity of pluc567 in HT29-Ad5-HIF-1α cells increased more than 5 fold compared with control cells, whereas the magnification was significantly degraded with pluc567-mut transfection (Fig 4C&4D). These results demonstrated that HIF-1α activated ZEB1 directly by binding to the HRE-3 site in the ZEB1 proximal promoter.

### ZEB1 is critical for HIF-1α-induced EMT and metastasis

To detect whether ZEB1 is required for HIF-1α-induced EMT and metastatic phenotype, we generated a plasmid with HIF-1α expressing and ZEB1 knockdown, and then packaged into adenovirus (Ad5-HIF-1α-shZEB1). Because the endogenous level of ZEB1 in HT29 cells was much lower than that in HCT116 cells (Fig 3C, right), which was consistent with their protein levels in our previous data [12]. Therefore, HCT116 cells were appointed to perform all of the ZEB1 knockdown experiments.

**Fig 5:**
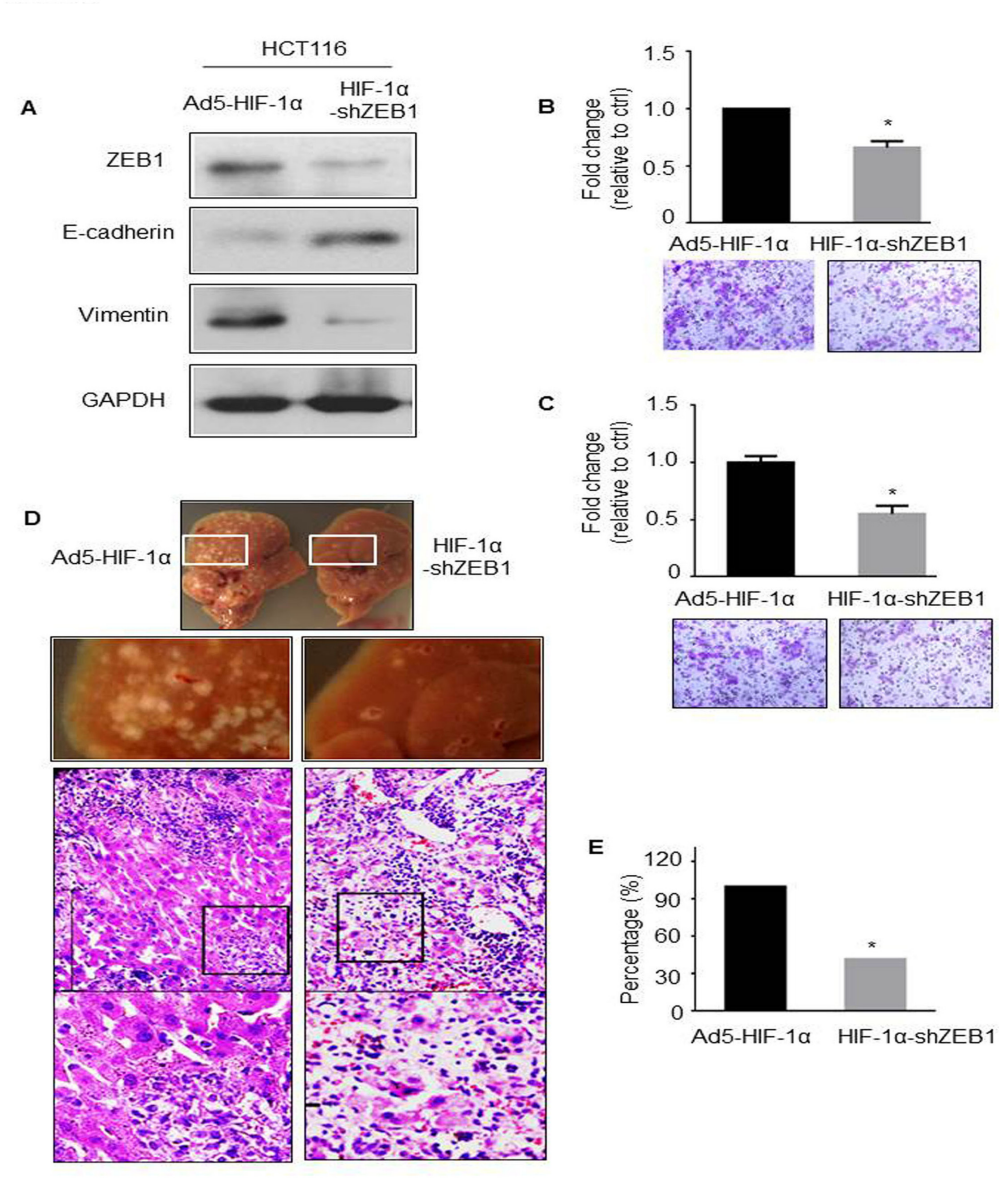
ZEB1 inhibition abolished HIF-1α-induced EMT and metastasis. (A) Western blot analysis of ZEB1, E-cadherin and Vimentin in HCT116-Ad5-HIF-1α or HCT116-HIF-1α-shZEB1 cells. GAPDH was used as the loading control. Invasion (B) and migration (C) assays were performed in the same two cell lines. The representative images of invaded cells in the inserts of transwell chambers were shown at original magnification = 200X. *P < 0.05. (D) Representative photographic pictures and H&E staining of liver of BALB/c mice 5 weeks after subsplenic injection of HCT116-Ad5-HIF-1α or HCT116-HIF-1α-shZEB1 cells. (E) Quantification of the average numbers of metastatic foci in the livers of mice (N = 5). *P < 0.05.

*In vitro*, HCT116 cells were transduced with Ad5-HIF-1α-shZEB1 and Ad5-HIF-1α, respectively. Western blot, invasion and migration assays were carried out after 48 hours. As shown in Fig 5A, E-cadherin expression was upregualted and Vimentin expression was downregulated accompanied with the knockdown of ZEB1. Consistently, the invasion and migration capacities were decreased by 34% and 45%, respectively. *In vivo*, we observed multiple tumor nodules on the surface of livers and H&E staining showed basophilic tumor regions in livers of mice injected with HCT116-Ad5-HIF-1α cells. In contrast, animals injected with HCT116-Ad5-HIF-1α-shZEB1 cells had dramatically decreased tumor burdens with a reduction in the number and size of residual tumor nests, accompanied with more massive necrosis with inflammation region (Fig 5D&5E).

### Expression of HIF-1α, ZEB1, E-cadherin and Vimentin in primary and metastatic CRC specimens

**Fig 6:**
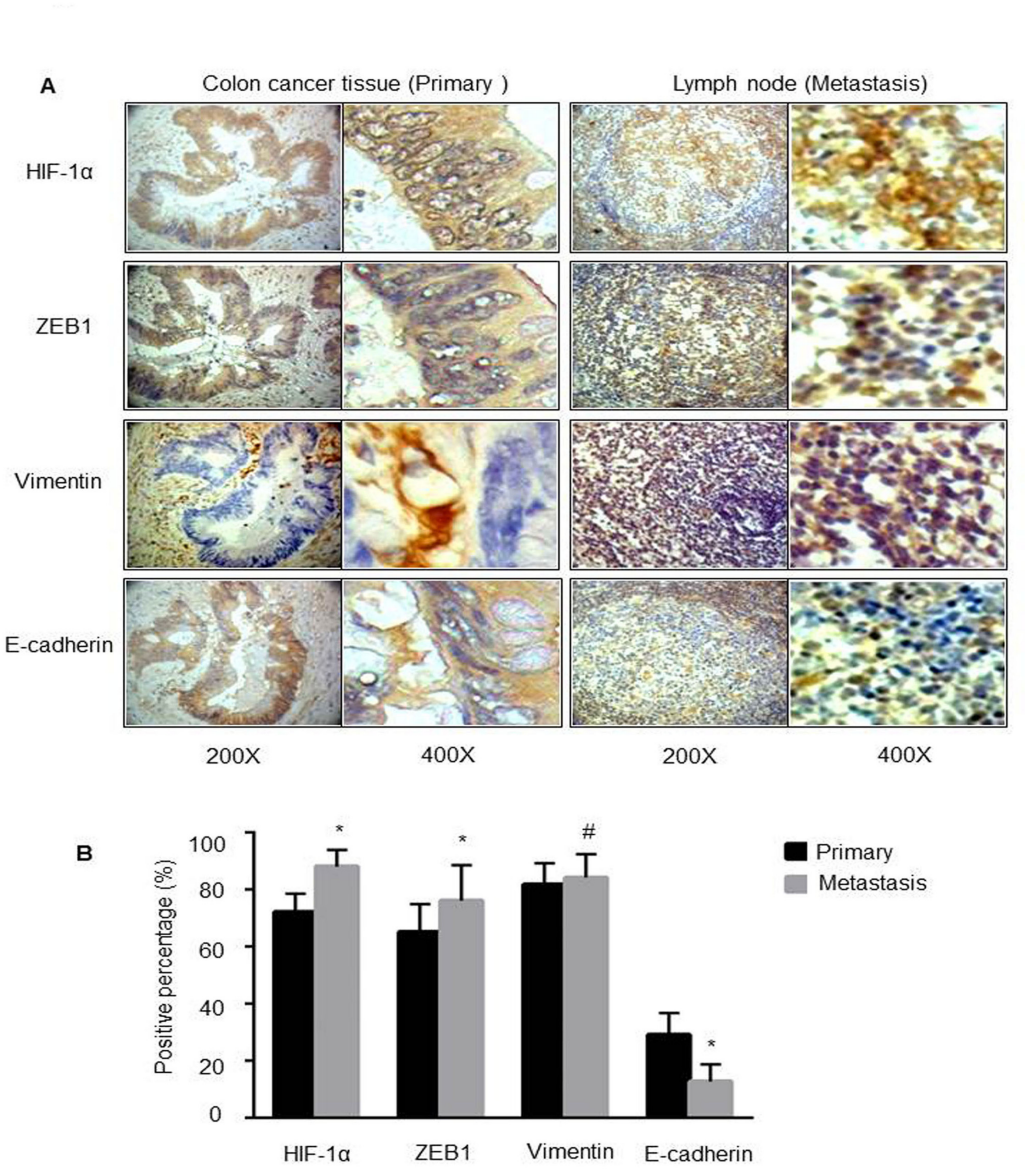
Expression statuses of HIF-1α, ZEB1, Vimentin and E-cadherin in tissue specimens. Representative images of IHC (A) and quantification of positive tumor cells (B) in both primary CRC specimens and the according metastatic lymph node. Original magnification, 200X. *P < 0.05 and ^#^ *P* > 0.05.

To evaluate the relationship among HIF-1α, ZEB1 and EMT key markers in clinical tissues, IHC staining was performed in 32 pairs of primary CRC specimens and metastatic lymph node (Fig 6A). The average percentage of positively stained cells to all tumor cells in each tissue was evaluated independently by two investigators as described in material and method. We found that the percentages of both HIF-1α- and ZEB1-positive CRC cells were more than 65%, while the percentage in metastatic lymph node was increased approximately to 86% for HIF-1α and 78% for ZEB1 (Fig 6B). At the same time, the level of Vimentin was also pretty high in both primary and metastatic tissues; although there was no significant difference between these two groups. For E-cadherin, the percentage of E-cadherin-positive tumor cells in primary CRC tissue was about 29%, whereas significantly decreased to 12.7% in metastatic group (Fig 6B). These results support our observations with the CRC cell lines that HIF-1α expression was positively associated with ZEB1 and Vimentin, and negatively associated with E-cadherin, and HIF-1α and ZEB1 may contribute differentially to EMT and metastasis.

## Discussion

One of the fundamental ways for cancer treatment is the understanding of molecular mechanisms for tumorigenesis and cancer metastasis. In this study, we revealed that ZEB1 is a downstream target of HIF-1α and has a critical role in EMT and metastatic phenotypes induced by overexpression of HIF-1α. We propose that HIF-1α directly binds to the promoter of ZEB1 and serves as its critical positive regulator, thereby promoting EMT and cancer metastasis. Our findings uncover a novel mechanism by which HIF-1α regulates tumor progression and invasion.

HIF-1α expression is commonly upregulated in a lot of malignant tumors, and many publications have reported the close correlation between HIF-1α expression and increased aggressiveness and higher metastatic capacity in ovarian, breast, lung, prostate, colon and pancreas carcinomas [11–14]. For molecular level, HIF-1α exerts its biological function through activating target genes such as Snail, Twist and TCF3, which are all associated with EMT and metastasis [15–18]. However, it is important to identity more unknown targets and to reveal the link between HIF-1α activation and other oncogene or tumor suppressors.

ZEB1, a pro-metastatic transcription factor, is involved in cancer progression and metastasis [19, 20]. Mechanistically, ectopic expression of ZEB1 is sufficient to downregulate E-cadherin and to induce EMT in breast cancer by binding to the conserved E-boxes in E-cadherin promoter. The inhibition function of ZEB1 on E-cadherin thus promoting EMT is also observed in xenograft models of CRC [8]. Furthermore, ZEB1 mediates claudin-1-regulated changes in cell invasion and anoikis in CRC [21]. Similar to ZEB1, Snail and Twist are also important EMT transcription factors by silencing E-cadherin through binding to the E-box element in E-cadherin promoter [22]. Snail is identified as a HIF-1α target gene in mouse [23]; Twist is directly regulated by HIF-1α in head and neck squamous cell carcinoma (HNSCC) [18, 24]. In other words, those also mean that the HIF-1α pathway can regulate E-cadherin repression, presumably via Snail or Twist. Hence, due to the similar abilities of HIF-1α and ZEB1 to induce EMT and the overlapping phenotypes of HIF-1α and ZEB1 in null mice, it is likely that these two genes are located in the same pathway to regulate cancer metastasis. Therefore, we hypothesized that there might be a strong interactive link between ZEB1 and HIF-1α. Indeed, we found that HIF-1α was able to bind ZEB1 promoter through HRE-3 and positively regulated ZEB1 transactivity. These findings also suggest that Snail, Twist and ZEB1, the three major EMT regulators may regulate EMT and metastasis with some very similar mechanisms. Interestingly, Peinado et al reported that Snail, Slug, ZEB1 and ZEB2 recruit specific chromatin-remodeling complexes supports a dynamic link between transcription repression and epigenetic genes silencing of E-cadherin during tumor progression and EMT [25].

In this study, we showed for the first time that HIF-1α expression directly regulated ZEB1, and positively correlated with the expression of ZEB1 and Vimentin, and inversely correlated with E-cadherin in primary and metastatic CRC tissue samples, which suggest co-expression of HIF-1α, ZEB1, Vimentin and decreased of E-cadherin may be used as a valuable marker to predict the metastastic potential of CRC patients. However, further investigations are also required to understand better the roles of HIF-1α, ZEB1, Vimentin and E-cadherin in CRC progression and malignancy and to determine how dysregulation of these proteins may alter response to therapeutic intervention.

In summary, our data support a model in which HIF-1α overexpression enhances ZEB1 transactivity and expression through directly binding to its promoter, leading to a dramatic loss of E-cadherin and Plakoglobin expressions, gain of the expression of Vimentin and N-cadherin, and increased cell invasion and migration. These findings provide a molecular basis for promotion of the invasive cancer phenotype by HIF-1α overexpression.
